# Hospital Staffs Should Be Paid

**Published:** 1898-08

**Authors:** 


### HOSPITAL STAFFS SHOULD BE PAID.

It is a sad fact, but none the less a fact well recognized by all of us, that the medical profession of to-day does not as a whole receive that respect which is due it as a learned and philanthropic profession. It is also true that, as a consequence of this defiency of respect the profession finds it all but impossible as a whole to secure the remuneration for its services which would be in keeping with that of the other professions, let alone the true value of the services to the community. This lack of respect is not so often spoken of among us, and yet each one of us sees more or less evidence of it every day. There is no need to specify the ways in which this is shown, as we all know them but too well. The cause and the remedy are the matters of importance. One great cause, if not the chief one, is, we believe, the lack of respect the profession shows for itself in bartering away its services for little or nothing and in submitting to freely presenting our knowledge to vast numbers of people. As an abstract humanitarian principle it is right for us to succor the sick poor without price, but as a sociologic act it is a grave question if our doing so is not a serious factor in promoting poverty, shiftlessness and weak dependence, as well as a great damage to ourselves. It is wholly and absolutely wrong and immoral, and should be considered unprofessional conduct, for any physician to give his services free of all charge to any hospital, dispensary or other corporation organized for the care of the sick and injured. If one of us chooses to give his services freely to a sick individual, all well and good, but it is a curious and sinful distinction that has arisen in our institutional work where nurses, superintendents, attendants and all other employees, except the physicians, whose work is the most important and essential, are paid for their labor. Charitable organizations should pay their physicians just as they do their nurses or their gardeners. There is no justification whatever for the present method of conducting these institutions. If the physicians attached to these institutions were paid as they should be we would hear no more of the superabundance of hospitals and dispensaries. Herein lies the true remedy for the "dispensary evil," which is being fought so hard now in the Eastern cities. Here also is the only remedy for the lack of respect shown the profession. How can a sensible and hard-headed public be expected to regard as rational beings men who deliberately throw away their best services, not for the sick poor, mind you, for we can do all that in our office, but for some corporation organized to care for the sick in the easiest and approved fashion—a corporation which expects to pay good wages to all its employees except those whose services are absolutely essential, its physicians. And why does not the corporation expect to pay its physicians? Simply because we are fools enough to think we can increase our private practice by borrowing something from the luster of the institution to which we are attached. It is purest mock-heroics to say we do it for charity, because it is not true, and because charity is far better done in another way. Yet the nurse is said to give her life, or some years of it, to "charity," but she gets her week's wages, as does the clergyman whose whole time is thus taken up. Charity organizations can just as well raise money to pay their physicians as to pay their nurses, and if this resulted in fewer such organizations neither the profession nor the deserving poor would be the losers thereby. Until the medical profession grasps this fact, which should be self-evident and not even need stating to be accepted as true, just so long will the public look upon us, and more correctly than should be, as a set of harmless idiots of some use to the sick but whose opinions are to be received with a raising of the eyebrows and a compassionate aside. In our own hand lies wholly our attaining that respect which all acknowledge is due our calling, and the abatement of the overabundance of hospitals and dipensaries, which increase the number of paupers and render more helpless those already such, which sap the vitality and self-respect of the profession, which cause nine-tenths of intraprofessional strife and "politics," which detract from the dignity of the profession, and which rob the old as well as the young physician of the cases and remuneration that are his by right. An awakening is at hand, and the present generation of physicians will live to see steps taken to at least initiate the correction of these evils.—*Editorial in Cleveland Journal of Medicine.*

